# EVALUATING A CONSUMER-DIRECTED HEALTH CARE PILOT FOR OLDER PEOPLE IN THE COMMUNITY

**DOI:** 10.1093/geroni/igz038.1844

**Published:** 2019-11-08

**Authors:** Amanda A Phelan, Alice Duggan, Deirdre O’Donnell, and Fealy Gerard

**Affiliations:** University College Dublin, Dublin, Ireland, Ireland

## Abstract

In an ageing society with a policy focus on community (Oireachtas 2017), providing choice and self-determination in home care provision for older people is fundamental. Consumer directed care has increased in popularity in many countries (Low, Yap & Brodaty, 2011). In 2016, planning began in Community Healthcare Organisation 3 to pilot a CDHC pilot for community based older people. This study presents the findings of this pilot project. The study used the Moore et al.’s (2015) evaluation approach. Data collection methods were via descriptive questionnaires with qualitative investigation of stakeholder experiences as well as an economic review. Findings demonstrated the need for additional clarity in understandings of the CDHC. Care recipients and caregivers did report satisfaction with care, however, it was the accessibility of care rather than choice that was considered important. There were also issues related to capacity to manage the CDHC as well as capacity to give a choice of providers, particularly in rural areas. Although, broadly cost-neutral, processes and structures also require additional alignment to enhance both effectiveness and efficiency. Empowering care recipients and enabling greater choice, flexibility and autonomy in care organisation and delivery was a central focus of the CDHC pilot. However, additional consumer awareness and programme enhancement is needed to fully realize the potential of the CDHC. Study recommendations focus on areas of increased clarity about the CDHC, implementation enhancement, complementarity and incorporation of CDHC with existing home support service provision arrangements

